# Developing a Predictive Model for Asthma-Related Hospital Encounters in Patients With Asthma in a Large, Integrated Health Care System: Secondary Analysis

**DOI:** 10.2196/22689

**Published:** 2020-11-09

**Authors:** Gang Luo, Claudia L Nau, William W Crawford, Michael Schatz, Robert S Zeiger, Emily Rozema, Corinna Koebnick

**Affiliations:** 1 Department of Biomedical Informatics and Medical Education University of Washington Seattle, WA United States; 2 Department of Research & Evaluation Kaiser Permanente Southern California Pasadena, CA United States; 3 Department of Allergy and Immunology Kaiser Permanente South Bay Medical Center Harbor City, CA United States; 4 Department of Allergy Kaiser Permanente Southern California San Diego, CA United States

**Keywords:** asthma, forecasting, machine learning, patient care management, risk factors

## Abstract

**Background:**

Asthma causes numerous hospital encounters annually, including emergency department visits and hospitalizations. To improve patient outcomes and reduce the number of these encounters, predictive models are widely used to prospectively pinpoint high-risk patients with asthma for preventive care via care management. However, previous models do not have adequate accuracy to achieve this goal well. Adopting the modeling guideline for checking extensive candidate features, we recently constructed a machine learning model on Intermountain Healthcare data to predict asthma-related hospital encounters in patients with asthma. Although this model is more accurate than the previous models, whether our modeling guideline is generalizable to other health care systems remains unknown.

**Objective:**

This study aims to assess the generalizability of our modeling guideline to Kaiser Permanente Southern California (KPSC).

**Methods:**

The patient cohort included a random sample of 70.00% (397,858/568,369) of patients with asthma who were enrolled in a KPSC health plan for any duration between 2015 and 2018. We produced a machine learning model via a secondary analysis of 987,506 KPSC data instances from 2012 to 2017 and by checking 337 candidate features to project asthma-related hospital encounters in the following 12-month period in patients with asthma.

**Results:**

Our model reached an area under the receiver operating characteristic curve of 0.820. When the cutoff point for binary classification was placed at the top 10.00% (20,474/204,744) of patients with asthma having the largest predicted risk, our model achieved an accuracy of 90.08% (184,435/204,744), a sensitivity of 51.90% (2259/4353), and a specificity of 90.91% (182,176/200,391).

**Conclusions:**

Our modeling guideline exhibited acceptable generalizability to KPSC and resulted in a model that is more accurate than those formerly built by others. After further enhancement, our model could be used to guide asthma care management.

**International Registered Report Identifier (IRRID):**

RR2-10.2196/resprot.5039

## Introduction

### Background

About 8.4% of people in the United States have asthma [1], which causes over 3000 deaths, around 500,000 hospitalizations, and over 2 million emergency department (ED) visits each year [1,2]. To improve patient outcomes and cut the number of asthma-related hospital encounters including ED visits and hospitalizations, predictive models are widely used to prospectively pinpoint high-risk patients with asthma for preventive care via care management. This is the case with health care systems such as the University of Washington Medicine, Kaiser Permanente Northern California [3], and Intermountain Healthcare, and with other health plans in 9 of 12 metropolitan communities [4]. Once a patient is identified as high risk and placed into a care management program, a care manager will call the patient periodically to assess asthma control, adjust asthma medications, and make appointments for needed care or testing. Successful care management can help patients with asthma obtain better outcomes, thereby avoiding up to 40% of their future hospital encounters [5-8].

A care management program has a limited service capacity and usually enrolls ≤3% of patients [9] with a given condition, which places a premium on enrolling at-risk patients. Therefore, the accuracy of the adopted predictive model (or lack thereof) puts an upper bound on the effectiveness of the program. Previously, several researchers have developed several models for projecting asthma-related hospital encounters in patients with asthma [3,10-22]. Each of these models would consider only a few features, miss more than half of patients who will have future asthma-related hospital encounters, and incorrectly project future asthma-related hospital encounters for many other patients with asthma [23]. These errors lead to suboptimal patient outcomes, including hospital encounters and unnecessary health care costs because of unneeded care management program enrollment. When building machine learning models on nonmedical data, people often follow the modeling guideline of checking extensive candidate features to boost model accuracy [24-27]. Adopting this modeling guideline to the medical domain, we recently constructed a machine learning model on Intermountain Healthcare data to project asthma-related hospital encounters in the following 12-month period in patients with asthma [23]. Compared with previous models, our model boosts the area under the receiver operating characteristic curve (AUC) by at least 0.049 to 0.859. Although this is encouraging, it remains unknown whether our modeling guideline is generalizable to other health care systems.

### Objectives

This study aims to assess the generalizability of our modeling guideline to Kaiser Permanente Southern California (KPSC). Similar to our Intermountain Healthcare model [23], our KPSC model uses administrative and clinical data to project asthma-related hospital encounters (ED visits and hospitalizations) in patients with asthma. The categorical dependent variable has 2 possible values—whether the patient with asthma will have asthma-related hospital encounters in the following 12-month period or not. This study describes the construction and evaluation of our KPSC model.

## Methods

The methods adopted in this study are similar to those used in our previous paper [23].

### Ethics Approval and Study Design

In this study, we performed a secondary analysis of computerized administrative and clinical data. This study was approved by the institutional review boards of the University of Washington Medicine and KPSC.

### Patient Population

**Figure 1:**
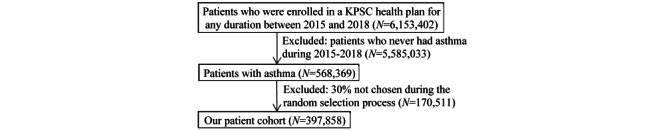
The patient cohort selection process. KPSC: Kaiser Permanente Southern California.

As shown in Figure 1, our patient cohort was based on patients with asthma who were enrolled in a KPSC health plan for any duration between 2015 and 2018. Owing to internal regulatory processes, the patient cohort was restricted to a random sample of 70.00% (397,858/568,369) of eligible patients. This sample size is the maximum that KPSC allows for sharing its data with an institution outside of Kaiser Permanente for research. As the largest integrated health care system in Southern California with 227 clinics and 15 hospitals, KPSC offers care to approximately 19% of Southern California residents [28]. A patient was deemed to have asthma in a particular year if the patient had one or more diagnosis codes of asthma (International Classification of Diseases [ICD], Tenth Revision [ICD-10]: J45.x; ICD, Ninth Revision [ICD-9]: 493.0x, 493.1x, 493.8x, 493.9x) recorded in the encounter billing database in that year [11,29,30]. The exclusion criterion was that the patient died during that year. If a patient had no diagnosis code of asthma in any subsequent year, the patient was deemed to have no asthma in that subsequent year.

### Prediction Target (the Dependent Variable)

For each patient identified as having asthma in a particular year, the outcome was whether the patient had any asthma-related hospital encounter in the following year. An asthma-related hospital encounter is an ED visit or hospitalization with asthma as the principal diagnosis (ICD-10: J45.x; ICD-9: 493.0x, 493.1x, 493.8x, 493.9x). For every patient with asthma, the patient’s data up to the end of every calendar year were used to project the patient’s outcome in the following year as long as the patient was deemed to have asthma in the previous year and was also enrolled in a KPSC health plan at the end of the previous year.

### Data Set

For the patients in our patient cohort, we used their entire electronically available patient history at KPSC. At KPSC, various kinds of information on its patients has been recorded in the electronic medical record system since 2010. In addition, we had electronic records of the patients’ diagnosis codes starting from 1981, regardless of whether they were stored in the electronic medical record system. From the research data warehouse at KPSC, we retrieved an administrative and clinical data set, including information regarding our patient cohort’s encounters and medication dispensing at KPSC from 2010 to 2018 and diagnosis codes at KPSC from 1981 to 2018. Owing to regulatory and privacy concerns, the data set is not publicly available.

### Features (Independent Variables)

We examined 2 types of candidate features—basic and extended. A basic feature and its corresponding extended features differ only in the year of the data used for feature computation. We considered 307 basic candidate features listed in Multimedia Appendix 1 [31]. Covering a wide range of characteristics, these basic candidate features were computed from the structured attributes in our data set. In Multimedia Appendix 1, unless the word *different* shows up, every mention of the number of a given type of item such as medications counts multiplicity. As defined in our previous paper [23], major visits for asthma include ED visits and hospitalizations with an asthma diagnosis code and outpatient visits with a primary diagnosis of asthma. Outpatient visits with a secondary but no primary diagnosis of asthma is regarded as minor visits for asthma.

Every input data instance to the model targets a unique (patient, index year) pair and is employed to forecast the patient’s outcome in the following year. For the (patient, index year) pair, the patient’s primary care provider (PCP), age, and home address were computed as of the end of the index year. The basic candidate features of history of bronchiolitis, the number of years since the first asthma-coded encounter in the data set, premature birth, family history of asthma, and the number of years since the first encounter for chronic obstructive pulmonary disease in the data set were computed using the data from 1981 to the index year. All of the allergy features and the features derived from the problem list were computed using the data from 2010 to the index year. One basic candidate feature was computed using the data in the index and preindex years: the proportion of patients who had asthma-related hospital encounters in the index year out of all of the patients of the patient’s PCP with asthma in the preindex year. The other 277 basic candidate features were computed using the data in the index year.

In addition to the basic candidate features, we also checked extended candidate features. Our Intermountain Healthcare model [23] was built using the extreme gradient boosting (XGBoost) machine learning classification algorithm [32]. As detailed in Hastie et al [33], XGBoost automatically computes the importance value of every feature as the fractional contribution of the feature to the model. Previously, we showed that ignoring those features with importance values <0.01 led to a little drop in model accuracy [23]. Using the basic candidate features and the model construction method described below, we built an initial XGBoost model on KPSC data. As a patient’s demographic features rarely change over time, no extended candidate feature was formed for any of the basic demographic features. For each basic candidate feature that was nondemographic, was computed on the data in the index year, and had an importance value 0.01 in the initial XGBoost model, we computed 2 related extended candidate features, one using the data in the preindex year and another using the data in the year that was 2 years before the index year. The only difference between the extended candidate features and the basic feature is the year of the data used for feature computation. For instance, for the basic candidate feature *number of ED visits in 2016*, the 2 related extended candidate features are the number of ED visits in 2015 and the number of ED visits in 2014. In brief, we formed extended candidate features for only those suitable and important basic candidate features. Our intuition is that among all possible ones that could be formed, these extended candidate features are most promising with regard to additional predictive power. For the other basic candidate features with lower importance values, those extended candidate features that could possibly be formed for them tend to have little extra predictive power and can be ignored. Given the finite data instances available for model training, this feature extending approach avoids a large rise in the number of candidate features, which may cause sample size issues. We considered all of the basic and extended candidate features when building our final predictive model.

### Data Analysis

#### Data Preparation

Peak expiratory flow values are available in our KPSC data set but not in the Intermountain Healthcare data set used in our previous paper [23]. On the basis of the upper and lower bounds given by a medical expert (MS) in our team, all peak expiratory flow values >700 were regarded as biologically implausible. Using this criterion and the same data preparation method adopted in our previous paper [23], we normalized data, identified biologically implausible values, and set them to missing. As the outcomes were from the following year and the extended candidate features were computed using the data from up to 2 years before the index year, our data set contained 6 years of effective data (2012-2017) over a total of 9 years (2010-2018). In clinical practice, a model is trained on historical data and then applied to future years’ data. To mirror this, the 2012 to 2016 data were used as the training set for model training. The 2017 data were employed as the test set to gauge model performance.

#### Performance Metrics

As shown in the formulas below and Table 1, we adopted 6 standard metrics to assess model performance: accuracy, specificity, sensitivity, negative predictive value (NPV), positive predictive value (PPV), and AUC.

Accuracy=(TP+TN)/(TP+TN+FP+FN),

Specificity=TN/(TN+FP),

Sensitivity=TP/(TP+FN),

Negative predictive value=TN/(TN+FN),

Positive predictive value=TP/(TP+FP).

We performed a 1000-fold bootstrap analysis [34] to compute the 95% CIs of these performance measures. We plotted the receiver operating characteristic (ROC) curve to show the tradeoff between sensitivity and specificity.

**Table 1:** The error matrix.

Outcome class	Asthma-related hospital encounters in the following year	No asthma-related hospital encounter in the following year
Projected asthma-related hospital encounters in the following year	TP^a^	FP^b^
Projected no asthma-related hospital encounter in the following year	FN^c^	TN^d^

^a^TP: true positive.

^b^FP: false positive.

^c^FN: false negative.

^d^TN: true negative.

#### Classification Algorithms

We employed Waikato Environment for Knowledge Analysis (WEKA) Version 3.9 [35] to build machine learning models. As a major open source toolkit for machine learning and data mining, WEKA integrates many classic feature selection techniques and machine learning algorithms. We examined the 39 native machine learning classification algorithms in WEKA, as shown in the web-based appendix of our previous paper [23] and the XGBoost classification algorithm [32] realized in the XGBoost4J package [36]. As an ensemble of decision trees, XGBoost implements gradient boosting in a scalable and efficient manner. As XGBoost takes only numerical features as its inputs, we converted every categorical feature to one or more binary features through one-hot encoding before giving the feature to XGBoost. We employed our previously developed automatic and efficient machine learning model selection method [37] and the 2012 to 2016 training data to automatically choose, among all of the applicable ones, the classification algorithm, feature selection technique, hyperparameter values, and data balancing method for managing imbalanced data. On average, our method runs 28 times faster and achieves an 11% lower model error rate than the Auto-WEKA automatic model selection method [37,38].

#### Assessing the Generalizability of our Intermountain Healthcare Model to KPSC

This study mainly assessed our modeling guideline’s generalizability to KPSC by using the KPSC training set to train several models and assessing their performance on the KPSC test set. In addition, we assessed our Intermountain Healthcare model’s [23] generalizability to KPSC. Using the Intermountain Healthcare data set and the top 21 features with an importance value computed by XGBoost ≥0.01, we formerly built a simplified Intermountain Healthcare model [23]. The simplified model retained almost all of the predictive power of our full Intermountain Healthcare model. Our KPSC data set included these 21 features but not all of the 142 features used in our full Intermountain Healthcare model. We assessed our simplified Intermountain Healthcare model’s performance on the KPSC test set twice, once after retraining the model on the KPSC training set and once using the model trained on the Intermountain Healthcare data set without retraining the model on the KPSC training set.

## Results

### Clinical and Demographic Characteristics of the Patient Cohorts

Every data instance targets a unique (patient, index year) pair. Multimedia Appendix 1 displays the clinical and demographic characteristics of our patient cohort during the time periods of 2012 to 2016 and 2017. The set of characteristics during 2012 to 2016 is similar to that during 2017. During 2012 to 2016 and 2017, 2.42% (18,925/782,762) and 2.13% (4353/204,744) of data instances were associated with asthma-related hospital encounters in the following year, respectively.

Table 2 shows for each clinical or demographic characteristic, the statistical test results on whether the data instances linking to future asthma-related hospital encounters and those linking to no future asthma-related hospital encounter had the same distribution. These 2 sets of data instances had the same distribution when the *P* value is ≥.05, and distinct distributions when the *P* value is <.05. In Table 2, all of the *P* values <.05 are marked in italics.

**Table 2:** For each clinical or demographic characteristic, the statistical test results on whether the data instances linking to future asthma-related hospital encounters and those linking to no future asthma-related hospital encounter had the same distribution.

Characteristics		*P* value for the 2012-2016 data	*P* value for the 2017 data
Age (years)		* <.001* ^a,b^	* <.001* ^a^
Gender		* <.001* ^c^	* .01* ^c^
Race		* <.001* ^c^	* <.001* ^c^
Ethnicity		* <.001* ^c^	* <.001* ^c^
Insurance category		* <.001* ^c^	* <.001* ^c^
Number of years since the first asthma-coded encounter in the data set		.78^a^	*.006* ^a^
**Asthma medication fill**			
	Inhaled corticosteroid	* <.001* ^c^	* <.001* ^c^
	Inhaled corticosteroid and long-acting beta-2 agonist combination	* <.001* ^c^	* <.001* ^c^
	Leukotriene modifier	* <.001* ^c^	* <.001* ^c^
	Long-acting beta-2 agonist	* <.001* ^c^	* <.001* ^c^
	Mast cell stabilizer	>.99^c^	>.99^c^
	Short-acting, inhaled beta-2 agonist	*<.001* ^c^	* <.001* ^c^
	Systemic corticosteroid	*<.001* ^c^	*<.001* ^c^
**Comorbidity**			
	Allergic rhinitis	* <.001* ^c^	* <.001* ^c^
	Anxiety or depression	* <.001* ^c^	* <.001* ^c^
	Bronchopulmonary dysplasia	* <.001* ^c^	>.99^c^
	Chronic obstructive pulmonary disease	* <.001* ^c^	* <.001* ^c^
	Cystic fibrosis	>.99^c^	.52^c^
	Eczema	*<.001* ^c^	*<.001* ^c^
	Gastroesophageal reflux	* <.001* ^c^	* <.001* ^c^
	Obesity	* <.001* ^c^	* <.001* ^c^
	Premature birth	* <.001* ^c^	* <.001* ^c^
	Sinusitis	.33^c^	.06^c^
	Sleep apnea	* .003* ^c^	* <.001* ^c^
Smoking status		* <.001* ^c^	* <.001* ^c^

^a^*P* values obtained by performing the Cochran-Armitage trend test [39].

^b^*P* values <.05 marked in italics.

^c^*P* values obtained by performing the chi-square two-sample test.

### Classification Algorithm and Features Used

Before building our final model, the importance values of the basic candidate features were computed once on our initial XGBoost model. This led to us examining 30 extended candidate features in addition to the 307 basic candidate features. With these 337 basic and extended candidates features as inputs, our automatic model selection method [37] picked the XGBoost classification algorithm [32]. As an ensemble of decision trees, XGBoost can handle missing feature values naturally. Our final predictive model was built using XGBoost, and the 221 features shown in descending order of importance value in Multimedia Appendix 1. The other features had no additional predictive power and were automatically dropped by XGBoost.

### Performance Measures of the Final KPSC Model

**Figure 2:**
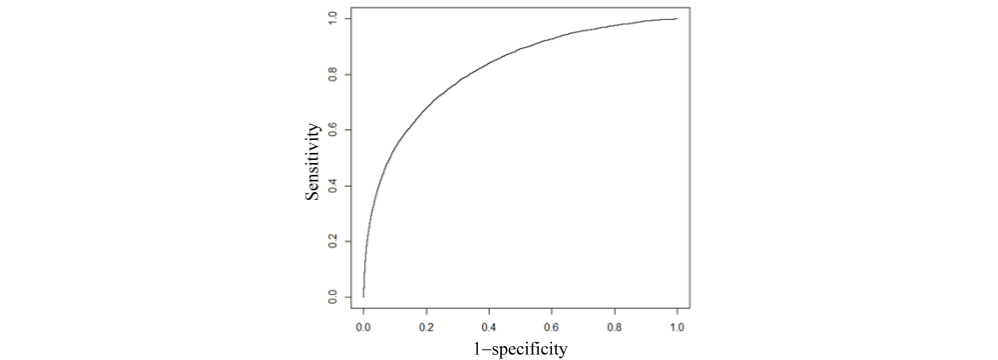
The receiver operating characteristic curve of our final predictive model.

On the KPSC test set, our final model achieved an AUC of 0.820 (95% CI 0.813-0.826). Figure 2 displays the ROC curve of our final model. Table 3 displays the performance measures of our final model when various top percentages of patients having the largest predicted risk were adopted as the cutoff point for performing binary classification. When this percentage was at 10.00% (20,474/204,744), our final model achieved an accuracy of 90.08% (184,435/204,744; 95% CI 89.95-90.21), a sensitivity of 51.90% (2259/4353; 95% CI 50.44-53.42), a specificity of 90.91% (182,176/200,391; 95% CI 90.78-91.03), a PPV of 11.03% (2259/20,474; 95% CI 10.59-11.46), and an NPV of 98.86% (182,176/184,270; 95% CI 98.81-98.91). Table 4 gives the corresponding error matrix of our final model.

When we excluded the extended candidate features and considered only the basic candidate features, the AUC of our model dropped to 0.809. Several basic candidate features, such as the number of years since the first asthma-coded encounter in the data set, needed over one year of past data to calculate. When we further excluded these multiyear candidate features and considered only those basic candidate features calculated on 1 year of past data, the model’s AUC dropped to 0.807.

Without precluding any feature from being considered, the model trained on data from both children (aged <18 years) with asthma and adults (aged ≥18 years) with asthma gained an AUC of 0.815 in children with asthma and an AUC of 0.817 in adults with asthma. In comparison, the model trained only on data from children with asthma gained an AUC of 0.811 in children with asthma. The model trained only on data from adults with asthma gained an AUC of 0.818 in adults with asthma.

If we adopted only the top 25 features shown in Multimedia Appendix 1 with an importance value ≥0.01 and removed the other 312 features, the model’s AUC dropped from 0.820 to 0.800 (95% CI 0.793-0.808). When the top 10.00% (20,474/204,744) of patients having the largest predicted risk were adopted as the cutoff point for doing binary classification, the model’s accuracy dropped from 90.08% (184,435/204,744) to 89.96% (184,185/204,744; 95% CI 89.83-90.08), sensitivity dropped from 51.90% (2259/4353) to 49.02% (2134/4353; 95% CI 47.71-50.55), specificity dropped from 90.91% (182,176/200,391) to 90.85% (182,051/200,391; 95% CI 90.72-90.97), PPV dropped from 11.03% (2259/20,474) to 10.42% (2134/20,474; 95% CI 10.03-10.86), and NPV dropped from 98.86% (182,176/184,270) to 98.80% (182,051/184,270; 95% CI 98.75-98.85).

**Table 3:** The performance measures of our final predictive model when various top percentages of patients having the largest predicted risk were adopted as the cutoff point for doing binary classification.

Top percentage of patients having the largest predicted risk (%)	Accuracy (N=204,744), n (%)	Sensitivity (N=4353), n (%)	Specificity (N=200,391), n (%)	PPV^a^			NPV^b^	
				n (%)	N	n (%)		N
1	199,732 (97.55)	694 (15.94)	199,038 (99.32)	694 (33.90)	2047	199,038 (98.19)		202,697
2	198,349 (96.88)	1026 (23.57)	197,323 (98.47)	1026 (25.06)	4094	197,323 (98.34)		200,650
3	196,831 (96.14)	1291 (29.66)	195,540 (97.58)	1291 (21.02)	6142	195,540 (98.46)		198,602
4	195,186 (95.33)	1492 (34.28)	193,694 (96.66)	1492 (18.22)	8189	193,694 (98.54)		196,555
5	193,472 (94.49)	1659 (38.11)	191,813 (95.72)	1659 (16.21)	10,237	191,813 (98.62)		194,507
6	191,717 (93.64)	1805 (41.47)	189,912 (94.77)	1805 (14.69)	12,284	189,912 (98.68)		192,460
7	189,919 (92.76)	1930 (44.34)	187,989 (93.81)	1930 (13.47)	14,332	187,989 (98.73)		190,412
8	188,124 (91.88)	2056 (47.23)	186,068 (92.85)	2056 (12.55)	16,379	186,068 (98.78)		188,365
9	186,267 (90.98)	2151 (49.41)	184,116 (91.88)	2151 (11.67)	18,426	184,116 (98.82)		186,318
10	184,435 (90.08)	2259 (51.90)	182,176 (90.91)	2259 (11.03)	20,474	182,176 (98.86)		184,270
15	174,902 (85.42)	2611 (59.98)	172,291 (85.98)	2611 (8.50)	30,711	172,291 (99.00)		174,033
20	165,253 (80.71)	2905 (66.74)	162,348 (81.02)	2905 (7.09)	40,948	162,348 (99.12)		163,796
25	155,491 (75.94)	3143 (72.20)	152,348 (76.03)	3143 (6.14)	51,186	152,348 (99.21)		153,558

^a^PPV: positive predictive value.

^b^NPV: negative predictive value.

**Table 4:** The error matrix of our final predictive model when the top 10.00% (20,474/204,744) of patients having the largest predicted risk were adopted as the cutoff point for doing binary classification.

Outcome class	Asthma-related hospital encounters in the following year	No asthma-related hospital encounter in the following year
Projected asthma-related hospital encounters in the following year	2259	18,215
Projected no asthma-related hospital encounter in the following year	2094	182,176

### Performance Measures of the Simplified Intermountain Healthcare Model

When applying our simplified Intermountain Healthcare model trained on the Intermountain Healthcare data set [23] to the KPSC test set without retraining the model on the KPSC training set, the model gained an AUC of 0.751 (95% CI 0.742-0.759). When the top 10.00% (20,474/204,744) of patients having the largest predicted risk were adopted as the cutoff point for doing binary classification, the model achieved an accuracy of 89.64% (183,531/204,744; 95% CI 89.51-89.77), a sensitivity of 41.51% (1807/4353; 95% CI 40.14-42.97), a specificity of 90.68% (181,724/200,391; 95% CI 90.55-90.81), a PPV of 8.83% (1807/20,474; 95% CI 8.44-9.23), and an NPV of 98.62% (181,724/184,270; 95% CI 98.57-98.67).

After using the KPSC training set to retrain our simplified Intermountain Healthcare model [23], the model gained on the KPSC test set an AUC of 0.779 (95% CI 0.772-0.787). When the top 10.00% (20,474/204,744) of patients having the largest predicted risk were adopted as the cutoff point for doing binary classification, the model achieved an accuracy of 89.85% (183,953/204,744; 95% CI 89.71-89.97), a sensitivity of 46.36% (2018/4353; 95% CI 44.89-47.84), a specificity of 90.79% (181,935/200,391; 95% CI 90.65-90.91), a PPV of 9.86% (2018/20,474; 95% CI 9.45-10.25), and an NPV of 98.73% (181,935/184,270; 95% CI 98.68-98.78).

## Discussion

### Principal Findings

We used KPSC data to develop a model to forecast asthma-related hospital encounters in the following 12-month period in patients with asthma. Table 5 shows that, compared with the models formerly built by others [3,10-22], our final KPSC model gained a higher AUC, that is, our modeling guideline of checking extensive candidate features to boost model accuracy exhibited acceptable generalizability to KPSC. After further enhancement to automatically explain its predictions [40,41] and to raise its accuracy, our model could be used to direct asthma care management to help improve patient outcomes and reduce health care costs.

Asthma affects adults and children differently. Our final model gained a lower AUC in children than in adults. Additional work is required to understand the difference and to boost the prediction accuracy in children.

We examined 337 basic and extended candidate features. Approximately 65.6% (221/337) of these were used in our final model. Many of the unused features were correlated with the outcome variable but provided no additional predictive power on the KPSC data set beyond those used in our final model.

In Multimedia Appendix 1, the 8 most important features and several others within the top 25 features reflect the loss of asthma control. This loss of asthma control could be because of the severity of the patient’s asthma. It could also relate to management practices, treatment nonadherence, or socioeconomic factors for which we had no data.

When using our simplified Intermountain Healthcare model [23] without retraining it on the KPSC training set, the model achieved an AUC of 0.751 on the KPSC test set. Despite being 0.069 lower than our final KPSC model’s AUC, this AUC is higher than the AUCs of many previous models for predicting hospitalization and ED visits in patients with asthma (Table 5). Therefore, we regard our simplified Intermountain Healthcare model to have acceptable generalizability to KPSC.

**Table 5:** Our final Kaiser Permanente Southern California model in comparison with several previous models for forecasting hospitalizations and emergency department visits in patients with asthma.

Model	Prediction target	Number of features the model used	Number of data instances	Classification algorithm	The undesirable outcome’s prevalence rate in the whole data set (%)	AUC^a^	Sensitivity (%)	Specificity (%)	PPV^b^ (%)	NPV^c^ (%)
Our final KPSC^d^ model	Asthma-related hospital encounters	221	987,506	XGBoost^e^	23,278 (2.36)	0.820	2259 (51.90)	182,176 (90.91)	2259 (11.03)	182,176 (98.86)
Our Intermountain Healthcare model [23]	Asthma-related hospital encounters	142	334,564	XGBoost	12,144 (3.63)	0.859	436 (53.69)	16,955 (91.93)	436 (22.65)	16,955 (97.83)
Miller et al [15]	Asthma-related hospital encounters	17	2821	Logistic regression	8.5	0.81	—^f^	—	—	—
Loymans et al [10]	Asthma exacerbation	7	611	Logistic regression	13	0.8	—	—	—	—
Lieu et al [3]	Asthma-related hospitalization	7	16,520	Proportional hazards regression	1.8	0.79	—	—	—	—
Schatz et al [11]	Asthma-related hospitalization in children	5	4197	Logistic regression	1.4	0.781	43.9	89.8	5.6	99.1
Yurk et al [17]	Lost day or asthma-related hospital encounters	11	4888	Logistic regression	54	0.78	77	63	82	56
Eisner et al [12]	Asthma-related ED^g^ visit	3	2415	Logistic regression	18.3	0.751	—	—	—	—
Forno et al [22]	Severe asthma exacerbation	17	615	Scoring	69.6	0.75	—	—	—	—
Schatz et al [11]	Asthma-related hospitalization in adults	3	6904	Logistic regression	1.2	0.712	44.9	87.0	3.9	99.3
Lieu et al [3]	Asthma-related ED visit	7	16,520	Proportional hazards regression	6.4	0.69	—	—	—	—
Eisner et al [12]	Asthma-related hospitalization	1	2858	Logistic regression	32.8	0.689	—	—	—	—
Sato et al [13]	Severe asthma exacerbation	3	78	Classification and regression tree	21	0.625	—	—	—	—
Schatz et al [20]	Asthma-related hospital encounters	4	14,893	Logistic regression	6.5	0.614	25.4	92.0	22.0	93.2
Lieu et al [19]	Asthma-related hospital encounters	4	7141	Classification and regression tree	6.9	—	49.0	83.6	18.5	—

^a^AUC: area under the receiver operating characteristic curve.

^b^PPV: positive predictive value.

^c^NPV: negative predictive value.

^d^KPSC: Kaiser Permanente Southern California.

^e^XGBoost: extreme gradient boosting.

^f^The original paper presenting the model did not report the performance measure.

^g^ED: emergency department.

### Comparison With Previous Work

Multiple researchers have built models to forecast ED visits and hospitalizations in patients with asthma [3,10-23]. Table 5 compares our final KPSC model with those models, which encompass all pertinent models covered in the systematic review of Loymans et al [18]. With the exception of our Intermountain Healthcare model [23], every model formerly built by others [3,10-22] gained a lower AUC than our final KPSC model. Instead of being for all patients with asthma, the model by Miller et al [15] targets adults with difficult-to-treat or severe asthma, 8.5% of whom had future asthma-related hospital encounters. The model by Loymans et al [10] predicts asthma exacerbations with a prevalence rate of 13%. These 2 prevalence rates of the undesirable outcome are much higher than that in our KPSC data set. In addition, the target patient population and the prediction target of these 2 models are not comparable with those in our KPSC model. Except for these 2 models, each of the other models formerly built by others had an AUC ≤0.79, which is at least 0.030 lower than that of our KPSC model.

Compared with other models, the model by Yurk et al [17] gained a larger PPV and sensitivity mainly because of the use of a distinct prediction target: hospital encounters or one or more days lost because of missed work or reduced activities for asthma. This prediction target was easier to predict, as it occurred in 54% of the patients with asthma. If the model by Yurk et al [17] were used to predict asthma-related hospital encounters that occurred with approximately 2% of the patients with asthma, we would expect the model to gain a lower sensitivity and PPV.

Excluding the model by Yurk et al [17], all of the other models formerly built by others had a sensitivity ≤49%, which is smaller than what our final KPSC model gained: 51.90% (2259/4353). Sensitivity provides, among all patients with asthma who will have future asthma-related hospital encounters, the proportion of patients that the model pinpoints. As the population of patients with asthma is large, for every 1% increase in the identified proportion of patients with asthma who would have future asthma-related hospital encounters, effective care management could help improve patient outcomes, thereby avoiding up to 7200 more ED visits and 1970 more hospitalizations in the United States annually [1,5-8].

The PPV depends substantially on the prevalence rate of undesirable outcomes [42]. In our KPSC test data set, 2.13% (4353/204,744) of patients with asthma had future asthma-related hospital encounters. When the top 10.00% (20,474/204,744) of patients having the largest predicted risk were adopted as the cutoff point for performing binary classification, the maximum possible PPV that a perfect model could obtain is 21.26% (4353/20,474). Our final KPSC model gained a PPV of 11.03% (2259/20,474), which is 51.90% (2259/4353) of the maximum possible PPV. In comparison, in our Intermountain Healthcare test data set, 4.22% of patients with asthma had future asthma-related hospital encounters [23]. Our Intermountain Healthcare model gained a PPV of 22.65% (436/1925) [23], which is 53.7% (436/812) of the maximum possible PPV that a perfect model could obtain. On a data set in which 6.5% of patients with asthma had future asthma-related hospital encounters, the model by Schatz et al [20] gained a PPV of 22.0%. On a data set in which 6.9% of patients with asthma had future asthma-related hospital encounters, the model by Lieu et al [19] gained a PPV of 18.5%. Except for these PPVs and the PPV of the model by Yurk et al [17], none of the previously reported PPVs was more than 5.6%.

Despite being built using the same modeling guideline, our final KPSC model gained a lower AUC than our Intermountain Healthcare model [23]. This is largely because the percentage of data instances in the test set linking to future asthma-related hospital encounters differs greatly at Intermountain Healthcare and at KPSC: 4.22% (812/19,256) versus 2.13% (4353/204,744), respectively. The rarer the undesirable outcome, the harder it is to accurately predict it.

The top features with an importance value ≥0.01 in our final KPSC model are similar to those in our Intermountain Healthcare model [23]. In both our final KPSC and our Intermountain Healthcare models, many top features involve asthma medications and previous ED visits. When building our Intermountain Healthcare model, we did not consider several basic candidate features. They turned out to be top features in our final KPSC model and impacted the importance values and ranks of the other top features there.

When building our Intermountain Healthcare model, we did not incorporate any extended candidate features. Several such features appeared as top features in our final KPSC model. Their inclusion boosted the model accuracy on our KPSC data set. It is possible that including extended candidate features could also boost the model accuracy on our Intermountain Healthcare data set. This could be explored in future work.

Schatz et al [20] showed that in 2 Southern California cities, 6.5% of patients with asthma at KPSC had asthma-related hospital encounters in 2000. In comparison, 2.08% (4353/208,959) of patients with asthma at KPSC had asthma-related hospital encounters in 2018. This suggests that compared with 2 decades ago, KPSC manages patients with asthma better now.

### Considerations About Potential Clinical Use

Although more accurate than those formerly built by others, our final KPSC model still gained a somewhat low PPV of 11.03% (2259/20,474). However, our model could be clinically useful:

1. A PPV of 11.03% (2259/20,474) is acceptable for pinpointing high-risk patients with asthma to apply low-cost preventive interventions. Examples of such interventions include giving the patient a peak flow meter for self-monitoring at home and showing the patient how to use it, instructing the patient on the correct use of an asthma inhaler, asking a nurse to follow up on the patient with extra phone calls, and training the patient to write a diary on environmental triggers.
2. As explained above, because of the low prevalence rate of the undesirable outcome used in this study, even a perfect model would gain a small PPV. For this outcome, sensitivity matters more than PPV for judging the model’s possible clinical impact. Our final KPSC model gained a higher sensitivity than all of the models that were formerly built by others and used a comparable prediction target.
3. To allocate care management resources, health care systems such as the University of Washington Medicine, Kaiser Permanente Northern California [3], and Intermountain Healthcare are using proprietary models whose performance measures are akin to those of the models previously built by others. Our final KPSC model is more accurate than these models.

Our final KPSC model used 221 features. Cutting this number could facilitate the clinical deployment of the model. In this regard, if one could bear a small drop in prediction accuracy, one could adopt the top features having an importance value of, for example, 0.01 or more and remove the others. The importance value of a feature changes across health care systems. Ideally, before deciding which features to keep, one should first compute the importance values of the features on a data set from the intended health care system.

Most of the attributes that we used to compute the features adopted in our final KPSC model, particularly the top features, are routinely collected by electronic medical record systems. For future work, to make it easy for other health care systems to reuse our final KPSC model, we can resort to the Observational Medical Outcomes Partnership (OMOP) common data model [43]. This data model and its linked standardized terminologies [44] standardize administrative and clinical attributes from at least 10 large US health care systems [45,46]. We can extend this data model to include the attributes that are used in our final KPSC model but missed by the original data model. We rewrite our feature construction and model building code based on the extended OMOP common data model and post our code and the related data schema on a public website. After converting its data into our extended OMOP common data model format based on this data schema, a health care system can rerun our code on its data to obtain a simplified version of our final KPSC model tailored to its data. Hopefully, most of the predictive power of our final KPSC model can be retained similar to what this study showed for our Intermountain Healthcare model.

It is difficult to interpret an XGBoost model employing many features globally, as is the case with many other involved machine learning models. As an interesting topic for future work, we plan to use our previously proposed method [40,41] to automatically explain our final KPSC model’s predictions for each patient with asthma.

Our final KPSC model was an XGBoost model [32]. When classifying 2 unbalanced classes, XGBoost employs a hyperparameter scale_pos_weight to balance their weights [47]. To maximize the AUC of our KPSC model, our automatic model selection method [37] changed scale_pos_weight from its default value to balance the 2 classes of having future asthma-related hospital encounters or not [48]. As a side effect, this shrank the model’s projected probabilities of having future asthma-related hospital encounters to a large extent and made them differ greatly from the actual probabilities [48]. This does not affect the identification of the top few percent of patients with asthma who have the largest projected risk to receive care management or other preventive interventions. We could keep scale_pos_weight at its default value of 1 and not balance the 2 classes. This would avoid the side effect but drop the model’s AUC from 0.820 to 0.817 (95% CI 0.810-0.824).

### Limitations

This study has 3 limitations, all of which provide interesting areas for future work:

1. In addition to those examined in this study, other features could also help raise model accuracy. Our KPSC data set does not include some potentially relevant features, such as characteristics of the patient’s home environment and features computed on the data gathered by monitoring sensors attached to the patient’s body. It would be worthwhile to identify new predictive features from various data sources.
2. Our study used only non-deep learning machine learning algorithms and structured data. Using deep learning and including features computed from unstructured clinical notes may further boost model accuracy [41,49].
3. Our study assessed our modeling guideline’s generalizability to only one health care system. It would be interesting to evaluate our modeling guideline’s generalizability to other health care systems, such as academic health care systems that have different properties from KPSC and Intermountain Healthcare. Compared with nonacademic health care systems, academic health care systems tend to care for sicker and more complex patients [50]. To perform such an evaluation, we are working on obtaining a data set of patients with asthma from the University of Washington Medicine [49].

### Conclusions

In its first generalizability assessment, our modeling guideline of examining extensive candidate features to help boost model accuracy exhibited acceptable generalizability to KPSC. Compared with the models formerly built by others, our KPSC model for projecting asthma-related hospital encounters in patients with asthma gained a higher AUC. At present, predictive models are widely used as a core component of a decision support tool to prospectively pinpoint high-risk patients with asthma for preventive care via care management. After further enhancement, our KPSC model could be used to replace the existing predictive models in the decision support tool for better directing asthma care management to help improve patient outcomes and reduce health care costs.

## Appendix

Multimedia Appendix 1 The basic candidate features, the clinical and demographic characteristics of our patient cohort, and the features employed in our final predictive model and their importance values.

## Abbreviations

AUC: area under the receiver operating characteristic curve
ED: emergency department
FN: false negative
FP: false positive
ICD: International Classification of Diseases
KPSC: Kaiser Permanente Southern California
NPV: negative predictive value
OMOP: Observational Medical Outcomes Partnership
PCP: primary care provider
PPV: positive predictive value
ROC: receiver operating characteristic
TN: true negative
TP: true positive
WEKA: Waikato Environment for Knowledge Analysis
XGBoost: extreme gradient boosting
