# Supplementary material for: Developing a Predictive Model for Asthma-Related Hospital Encounters in Patients With Asthma in a Large, Integrated Health Care System: Secondary Analysis
Source: JMIR Med Inform. 2020 Nov 9;8(11):e22689. doi: 10.2196/22689 (PMC7683251; doi:10.2196/22689)
Supplement: Multimedia Appendix 1 [file medinform_v8i11e22689_app1.pdf]

Appendix

Table 1. The basic candidate features.

Feature category	Features
Patient demographics features	Gender; age; ethnicity (Hispanic or non-Hispanic); race; marital status (single, married, partnered, separated, widowed, or divorced); and language.
Features giving properties of the area with the five-digit zip code of the patient's home address	The area's percentage of Hispanic black population; percentage of non-Hispanic black population; percentage of Hispanic white population; percentage of non-Hispanic white population; percentage of Hispanic American Indian and Alaska native population; percentage of non-Hispanic American Indian and Alaska native population; percentage of Hispanic Asian population; percentage of non-Hispanic Asian population; percentage of Hispanic native Hawaiian or other Pacific islander population; percentage of non-Hispanic native Hawaiian or other Pacific islander population; percentage of Hispanic other-race population; percentage of non-Hispanic other-race population; percentage of Hispanic population of two or more races; percentage of non-Hispanic population of two or more races; household income level like the median household income; and the proportion having asthma-related hospital encounters out of all patients with asthma in the area. Except for the last one, all of these features were derived from 2010 census data.
Features giving properties of the census block group where the patient lives	The block group's percentage of population 25 and older with less than 9th grade education; percentage of population 25 and older with 9th-12th grade education; percentage of population 25 and older with a high school diploma; percentage of population 25 and older with college education and no degree; percentage of population 25 and older with an associate's degree; percentage of population 25 and older with a bachelor's degree; and percentage of population 25 and older with a graduate or professional degree. All of these features were computed on the five-year rolling averages from the US Census 2013 American Community Survey.
Features on laboratory tests	# of laboratory tests; # of days since taking the last laboratory test; # of laboratory tests with abnormal results; the maximum percentage of blood eosinophils; the maximum blood eosinophil count; whether an immunoglobulin E (IgE) test was done; whether the maximum total serum IgE level is abnormally high; and the maximum total serum IgE level.
Features about standard vital signs	The average diastolic blood pressure; the maximum diastolic blood pressure; the average systolic blood pressure; the maximum systolic blood pressure; the average heart rate; the maximum heart rate; the average respiratory rate; the maximum respiratory rate; the average temperature; the maximum temperature; the average peripheral capillary oxygen saturation (SpO_2); the minimum SpO_2; the average peak expiratory flow; the minimum peak expiratory flow; the average Z-score for length-for-age; the average Z-score for weight-for-age; the average Z-score for weight-for-length; the maximum body mass index (BMI); the relative change of BMI = (the last noted BMI / the first noted BMI - 1) × 100%; and the relative change of weight = (the last noted weight / the first noted weight - 1) × 100%.
Exercise vital sign features	The average # of days per week the patient exercises; and the average # of minutes per week the patient exercises.
Diagnosis-related features derived from ICD-10 and ICD-9 diagnosis codes only	# of ICD-10 and ICD-9 diagnosis codes; # of years since the first asthma-coded encounter in the data set; # of asthma diagnoses; # of primary or principal asthma diagnoses; whether the last asthma diagnosis is a primary or a principal one; the last asthma diagnosis' severity; the highest severity of all of the asthma diagnoses; # of diagnoses of asthma with (acute) exacerbation; # of diagnoses of asthma with status asthmaticus; the last asthma diagnosis' exacerbation severity (uncomplicated, exacerbation, or asthmaticus); the highest exacerbation severity of all of the asthma diagnoses; # of days since having the last asthma diagnosis; # of days since having the last diagnosis of asthma with (acute) exacerbation or status asthmaticus; family history of asthma; chronic obstructive pulmonary disease; # of years since the first encounter for chronic obstructive pulmonary disease in the data set; allergic rhinitis; ischemic heart disease; esophagitis; gastroesophageal reflux; anxiety or depression; sleep apnea; eczema; gastrostomy tube; obesity; Alzheimer's or Parkinson's disease; upper respiratory tract infection; bronchopulmonary dysplasia; history of bronchiolitis; increased tone; decreased tone; cystic fibrosis; premature birth; pneumonia; immunoglobulin A (IgA) deficiency; vocal cord dysfunction; anaphylaxis; psoriasis; cirrhosis; vasculitis; gastrointestinal bleeding;

	inflammatory bowel disease; breathing abnormality like dyspnea; gastrointestinal obstruction; mental disorder; vitamin D deficiency; pregnancy; myocardial infarction; folate deficiency; peripheral vascular disease; congestive heart failure; dementia; cerebrovascular disease; peptic ulcer disease; rheumatic disease; substance use; diabetes with chronic complication; diabetes without chronic complication; mild liver disease; renal disease; hemiplegia or paraplegia; moderate or severe liver disease; acquired immunodeficiency syndrome; metastatic solid tumor; malignancy; whether the patient had an asthma action plan; and # of diagnoses of noncompliance with medication regimen.
Diagnosis-related features derived collectively from Current Procedural Terminology (CPT) and Healthcare Common Procedure Coding System (HCPCS) procedure codes, and ICD-10 and ICD-9 diagnosis codes	Sinusitis; and cataract.
Diagnosis-related feature derived collectively from ICD-10 and ICD-9 procedure codes, and ICD-10 and ICD-9 diagnosis codes	Tracheostomy.
Features derived from the problem list	# of active problems; # of active problems of asthma; # of active problems of asthma with (acute) exacerbations; # of active problems of wheezing; # of active problems of obesity; # of active problems of sleep apnea; # of active problems of atherosclerosis; # of active problems of hyperlipidemia; # of active problems of congestive heart failure; # of active problems of chronic obstructive pulmonary disease; # of active problems of hypertension; # of active problems of diabetes; # of active problems of gastroesophageal reflux disease; # of active problems of rhinitis; # of active problems of anxiety/depression; # of active problems on smoking; # of active problems on care management; and the priority of the most recent active problem of asthma.
Features derived from the medical history	# of medical history diagnosis codes; and # of medical history diagnosis codes of asthma.
Features on medications	# of medication orders; total copay for medications; total # of medications ordered; total # of different medications ordered; total # of medications filled; total # of units of medications filled; the asthma medication ratio [50]; # of asthma medication orders; total copay for asthma medications; total # of asthma medications ordered; total # of different medications in all of the asthma medication orders; total # of asthma medications filled; total # of units of asthma medications filled; # of days since the last use of asthma medications; # of asthma medications used on the last day; total # of short-acting beta-2 agonists ordered; total # of units of short-acting beta-2 agonists filled; # of fills of short-acting beta-2 agonists; # of days since the last use of short-acting beta-2 agonists; total # of systemic corticosteroids ordered; total # of units of systemic corticosteroids filled; # of fills of systemic corticosteroids; # of days since the last use of systemic corticosteroids; # of asthma reliever orders; total # of asthma relievers filled; total # of asthma relievers ordered; total # of different asthma relievers ordered; total # of units of asthma relievers filled; # of days since the last use of asthma relievers; # of fills of asthma reliever medications that are neither systemic corticosteroids nor short-acting beta-2 agonists; total # of units of asthma reliever medications filled that are neither systemic corticosteroids nor short-acting beta-2 agonists; # of days since the last use of asthma controllers; # of days of supply of the last asthma controller fill; the strength of each dose of the last asthma controller fill; # of asthma controller orders; total # of asthma controllers filled; total # of asthma controllers ordered; total # of different asthma controllers ordered; total # of units of asthma controllers filled; total # of days of gap in asthma controller use; total # of inhaled corticosteroids ordered; total # of units of inhaled corticosteroids filled; # of fills of inhaled corticosteroids; total dose of inhaled corticosteroids; # of days since the last use of inhaled corticosteroids; total # of mast cell stabilizers ordered; total # of units of mast cell stabilizers

	filled; # of fills of mast cell stabilizers; whether spacer was used; whether nebulizer was used; # of nebulizer medication orders; total # of fills of nebulizer medications; # of days since the last use of nebulizer medications; total # of nebulizer medications ordered; total # of different nebulizer medications ordered; and total # of units of nebulizer medications filled.
Features on insurances	The patient's insurance category (commercial, exchange, public, self-paid plan, or other) on the last day.
Features on the patient's visit types	# of visits; # of outpatient visits; # of outpatient visits to the patient's PCP; # of outpatient visits having a primary diagnosis of asthma; # of ED visits; # of ED visits on asthma; the average length of stay of an ED visit; the last ED visit's length of stay; # of hospitalizations; the average length of a hospitalization; the total length of all of the hospitalizations; # of hospitalizations, ED visits, and outpatient visits; whether the patient had any hospitalization, ED visit, or outpatient visit on asthma; # of virtual visits; # of virtual visits by email; # of virtual visits by phone; # of urgent care visits; # of urgent care visits for asthma; # of other types of visits (e.g., home health) that are not outpatient visits, ED visits, hospitalizations, or virtual visits; # of admissions to intensive care; # of major visits for asthma; the last visit's admission type (emergency, urgent, elective, or trauma); the last visit's type (outpatient visit, ED visit, hospitalization, virtual, or other); # of visits on asthma care management; and the most emergent admission type of all of the visits.
Features about appointment scheduling and visit status	# of cancelled appointments; # of no shows; the day of the week of the last ED visit's admission time; the last visit's discharge disposition location (left against medical advice, home, or other non-home location); # of times the patient left against medical advice; for the last visit, the time from making the request to the actual visit indicating the request's urgency; among all of the visits, the shortest time from making the request to the actual visit; # of visits with same day appointments; # of days since the last hospitalization; whether the last hospitalization was through the ED; # of days since the last ED visit; # of days since the last ED visit on asthma; # of days since the last outpatient visit; # of days since the last outpatient visit on asthma; # of days since the last virtual visit; # of days since the last other type of visit that is not an outpatient visit, an ED visit, a hospitalization, or a virtual visit; the average length of appointment of an outpatient visit with asthma as the primary diagnosis; and the average length of appointment of an outpatient visit.
Features showing the patient's care continuity and access to KPSC resources	# of different EDs the patient visited; # of different PCPs of the patient; # of different providers seen in outpatient visits; # of different asthma medication prescribers; # of different medication prescribers; and whether the patient had access to kp.org.
Features on procedures	# of ICD-10 and ICD-9 procedure codes; mechanical ventilation indicated by ICD-10 and ICD-9 procedure codes; # of CPT/HCPCS procedure codes; # of HCPCS procedure codes of home oxygen therapy; # of CPT/HCPCS procedure codes of influenza vaccination; # of CPT procedure codes of the fractional exhaled nitric oxide (FeNO) test; and # of CPT procedure codes of pulmonary function tests.
Allergy features	# of allergies of the patient; indicator of drug or material allergy; the highest severity of the drug or material allergies the patient had; indicator of environmental allergy; the highest severity of the environmental allergies the patient had; indicator of food allergy; and the highest severity of the food allergies the patient had.
Feature on clinical assessments	The average asthma control test score; the minimum asthma control test score; whether asthma control test was done on the patient; the maximum Patient Health Questionnaire-9 (PHQ-9) total score; the average PHQ-9 total score; and whether PHQ-9 assessment was done on the patient. The asthma control test is used to assess the level of asthma control. A larger asthma control test score reflects better asthma control. The PHQ-9 is employed to diagnose, screen, measure the severity of, and monitor depression. A larger PHQ-9 total score reflects more severe depression.
Features on pulmonary function tests	The average pre-bronchodilator forced expiratory volume in 1 second / forced vital capacity (FEV1/FVC) ratio; the minimum pre-bronchodilator FEV1/FVC ratio; the average post-bronchodilator FEV1/FVC ratio; the minimum post-bronchodilator FEV1/FVC ratio; the average pre-bronchodilator FEV1% predicted; the minimum pre-bronchodilator FEV1% predicted; the average post-bronchodilator FEV1% predicted; the minimum post-

	bronchodilator FEV1% predicted; and whether any pulmonary function test was performed on the patient.
Features on social behavior history	Whether the patient drank alcohol based on the last record; whether the patient was ever recorded of drinking alcohol; # of fluid ounces of alcohol the patient drank per week based on the last record; the average # of fluid ounces of alcohol the patient drank per week across all of the records; # of alcohol drinks the patient had per week based on the last record; the average # of alcohol drinks the patient had per week across all of the records; whether the patient was a smoker based on the last record; whether the patient was a former smoker based on the last record; # of packs of cigarettes the patient smoked per day based on the last record; the average # of packs of cigarettes the patient smoked per day across all of the records; # of years for which the patient had smoked based on the last record; whether the patient used any illicit drug based on the last record; whether the patient was ever recorded of using an illicit drug; # of times per week the patient used illicit drugs based on the last record; and the average # of times per week the patient used illicit drugs across all of the records.
Provider features	The patient's current PCP is defined as the patient's PCP known at the last clinic visit. The PCP features include: the PCP's age; whether the patient and the PCP are of the same gender; # of years for which the PCP had practiced at KPSC; # of patients of the PCP; # of patients with asthma of the PCP; total # of opioid prescriptions the PCP ordered for the PCP's patients; total # of antibiotic prescriptions the PCP ordered for the PCP's patients; total # of oral steroid prescriptions the PCP ordered for the PCP's patients; and among all of the PCP's patients with asthma in the pre-index year, the proportion who had asthma-related hospital encounters in the index year.

Table 2. Clinical and demographic characteristics of our patient cohort.

Characteristic	Time period					
	2012-2016			2017		
	Data instances (n=782,762), n (%)	Data instances linking to asthma-related hospital encounters in the following year (n=18,925), n (%)	Data instances linking to no asthma-related hospital encounter in the following year (n=763,837), n (%)	Data instances (n=204,744), n (%)	Data instances linking to asthma-related hospital encounters in the following year (n=4353), n (%)	Data instances linking to no asthma-related hospital encounter in the following year (n=200,391), n (%)
**Age (years)**						
<6	53,744 (6.87)	3041 (16.07)	50,703 (6.64)	11,834 (5.78)	610 (14.01)	11,224 (5.60)
6 to <18	193,622 (24.74)	5039 (26.63)	188,583 (24.69)	44,868 (21.91)	1012 (23.25)	43,856 (21.89)
18 to 65	424,446 (54.22)	8557 (45.22)	415,889 (54.45)	112,021 (54.71)	2052 (47.14)	109,969 (54.88)
≥65	110,950 (14.17)	2288 (12.09)	108,662 (14.23)	36,021 (17.59)	679 (15.60)	35,342 (17.64)
**Gender**						
Male	328,762 (42.00)	8335 (44.04)	320,427 (41.95)	84,249 (41.15)	1871 (42.98)	82,378 (41.11)
Female	454,000 (58.00)	10,590 (55.96)	443,410 (58.05)	120,495 (58.85)	2482 (57.02)	118,013 (58.89)
**Race**						
American Indian or Alaska native	3831 (0.49)	86 (0.45)	3745 (0.49)	1018 (0.50)	31 (0.71)	987 (0.49)
Asian	70,063 (8.95)	1282 (6.77)	68,781 (9.00)	18,874 (9.22)	319 (7.33)	18,555 (9.26)
Black or African American	115,851 (14.80)	4982 (26.33)	110,869 (14.51)	27,939 (13.65)	1075 (24.70)	26,864 (13.41)
Native Hawaiian or other Pacific Islander	7922 (1.01)	230 (1.22)	7692 (1.01)	1952 (0.95)	42 (0.96)	1910 (0.95)
White	487,582 (62.29)	10,040 (53.05)	477,542 (62.52)	126,816 (61.94)	2302 (52.88)	124,514 (62.14)
Unknown or unreported	97,513 (12.46)	2305 (12.18)	95,208 (12.46)	28,145 (13.75)	584 (13.42)	27,561 (13.75)
**Ethnicity**						

Hispanic	307,371 (39.27)	8131 (42.96)	299,240 (39.18)	80,021 (39.08)	1868 (42.91)	78,153 (39.00)
Non-Hispanic	460,372 (58.81)	10,577 (55.89)	449,795 (58.89)	119,211 (58.22)	2410 (55.36)	116,801 (58.29)
Unknown or unreported	15,019 (1.92)	217 (1.15)	14,802 (1.94)	5512 (2.69)	75 (1.72)	5437 (2.71)
**Insurance**						
Commercial (employer-paid)	532,412 (68.02)	11,311 (59.77)	521,101 (68.22)	130,144 (63.56)	2420 (55.59)	127,724 (63.74)
Exchange (marketplace)	39,785 (5.08)	735 (3.88)	39,050 (5.11)	17,946 (8.77)	269 (6.18)	17,677 (8.82)
Public	223,789 (28.59)	7469 (39.47)	216,320 (28.32)	66,631 (32.54)	1904 (43.74)	64,727 (32.30)
Self-paid plan	106,703 (13.63)	2224 (11.75)	104,479 (13.68)	34,405 (16.80)	647 (14.86)	33,758 (16.85)
Other	271,328 (34.66)	6064 (32.04)	265,264 (34.73)	84,783 (41.41)	1675 (38.48)	83,108 (41.47)
High deductible plan	81,819 (10.45)	1426 (7.54)	80,393 (10.52)	25,003 (12.21)	356 (8.18)	24,647 (12.30)
**Number of years since the first asthma-coded encounter in the data set**						
≤3	331,913 (42.40)	8006 (42.30)	323,907 (42.41)	85,843 (41.93)	1737 (39.90)	84,106 (41.97)
>3	450,849 (57.60)	10,919 (57.70)	439,930 (57.59)	118,901 (58.07)	2616 (60.10)	116,285 (58.03)
**Asthma medication fill**						
Inhaled corticosteroid	336,997 (43.05)	11,841 (62.57)	325,156 (42.57)	80,806 (39.47)	2586 (59.41)	78,220 (39.03)
Inhaled corticosteroid and long-acting beta-2 agonist combination	92,822 (11.86)	3975 (21.00)	88,847 (11.63)	29,731 (14.52)	1151 (26.44)	28,580 (14.26)
Leukotriene modifier	89,424 (11.42)	4125 (21.80)	85,299 (11.17)	28,095 (13.72)	1099 (25.25)	26,996 (13.47)
Long-acting beta-2 agonist	35,270 (4.51)	1694 (8.95)	33,576 (4.40)	11,810 (5.77)	467 (10.73)	11,343 (5.66)
Mast cell stabilizer	20 (0.00)	0 (0.00)	20 (0.00)	0 (0.00)	0 (0.00)	0 (0.00)
Short-acting, inhaled beta-2 agonist	553,684 (70.73)	16,242 (85.82)	537,442 (70.36)	140,819 (68.78)	3742 (85.96)	137,077 (68.40)
Systemic corticosteroid	247,083 (31.57)	10,837 (57.26)	236,246 (30.93)	67,475 (32.96)	2597 (59.66)	64,878 (32.38)
**Comorbidity**						
Allergic rhinitis	168,709 (21.55)	4673 (24.69)	164,036 (21.48)	40,933 (19.99)	1084 (24.90)	39,849 (19.89)
Anxiety or depression	164,950 (21.07)	4231 (22.36)	160,719 (21.04)	47,300 (23.10)	1124 (25.82)	46,176 (23.04)
Bronchopulmonary dysplasia	263 (0.03)	22 (0.12)	241 (0.03)	30 (0.01)	1 (0.02)	29 (0.01)
Chronic obstructive pulmonary disease	28,387 (3.63)	999 (5.28)	27,388 (3.59)	7591 (3.71)	285 (6.55)	7306 (3.65)
Cystic fibrosis	138 (0.02)	3 (0.02)	135 (0.02)	42 (0.02)	2 (0.05)	40 (0.02)
Eczema	85,369 (10.91)	2944 (15.56)	82,425 (10.79)	21,159 (10.33)	638 (14.66)	20,521 (10.24)
Gastroesophageal reflux	103,958 (13.28)	2778 (14.68)	101,180 (13.25)	33,259 (16.24)	797 (18.31)	32,462 (16.20)
Obesity	176,442 (22.54)	4776 (25.24)	171,666 (22.47)	49,738 (24.29)	1190 (27.34)	48,548 (24.23)
Premature birth	17,297 (2.21)	690 (3.65)	16,607 (2.17)	4513 (2.20)	132 (3.03)	4381 (2.19)
Sinusitis	115,173 (14.71)	2832 (14.96)	112,341 (14.71)	29,882 (14.59)	680 (15.62)	29,202 (14.57)
Sleep apnea	21,040 (2.69)	575 (3.04)	20,465 (2.68)	13,144 (6.42)	333 (7.65)	12,811 (6.39)
**Smoking status**						
Current smoker	157,288 (20.09)	4170 (22.03)	153,118 (20.05)	40,093 (19.58)	973 (22.35)	39,120 (19.52)
Former smoker	136,326 (17.42)	2870 (15.17)	133,456 (17.47)	36,743 (17.95)	717 (16.47)	36,026 (17.98)
Never smoker or unknown	489,148 (62.49)	11,885 (62.80)	477,263 (62.48)	127,908 (62.47)	2663 (61.18)	125,245 (62.50)

Table 3. The features employed in our final predictive model and their importance values.

Rank	Feature	Importance computed as the feature's fractional contribution to the model
1	Total # of units of nebulizer medications filled	0.0819
2	# of asthma reliever orders	0.0794
3	Total # of asthma relievers ordered	0.0655
4	# of days since having the last diagnosis of asthma with (acute) exacerbation or status asthmaticus	0.0541
5	# of ED visits on asthma	0.0437
6	# of nebulizer medication orders	0.0336
7	# of ED visits	0.0328
8	# of ED visits on asthma in the pre-index year	0.0279
9	Age	0.0264
10	# of primary or principal asthma diagnoses	0.0230
11	# of ED visits in the pre-index year	0.0204
12	Total # of asthma relievers filled	0.0201
13	Total # of units of nebulizer medications filled in the pre-index year	0.0184
14	# of major visits for asthma in the pre-index year	0.0170
15	The highest exacerbation severity of all of the asthma diagnoses in the pre-index year	0.0169
16	# of nebulizer medication orders in the pre-index year	0.0157
17	# of days since the last ED visit on asthma	0.0153
18	# of ED visits on asthma in the pre-pre-index year	0.0143
19	The day of the week of the last ED visit's admission time	0.0142
20	The highest exacerbation severity of all of the asthma diagnoses	0.0139
21	Total # of short-acting beta-2 agonists ordered	0.0127
22	# of ED visits in the pre-pre-index year	0.0125
23	Whether the patient is black or African American	0.0124
24	Total copay for medications in the pre-pre-index year	0.0101
25	# of primary or principal asthma diagnoses in the pre-index year	0.0101
26	# of asthma reliever orders in the pre-index year	0.0097
27	# of active problems of asthma	0.0089
28	# of asthma diagnoses	0.0087
29	# of major visits for asthma	0.0084
30	# of days since the last use of asthma relievers	0.0078
31	Total copay for medications	0.0066
32	The highest exacerbation severity of all of the asthma diagnoses in the pre-pre-index year	0.0063
33	# of primary or principal asthma diagnoses in the pre-pre-index year	0.0063
34	Total # of units of nebulizer medications filled in the pre-pre-index year	0.0060
35	Total # of units of asthma relievers filled in the pre-index year	0.0057
36	Total # of units of medications filled	0.0055
37	# of virtual visits by email	0.0055
38	Whether the patient had access to kp.org	0.0052
39	# of no shows	0.0051
40	# of active problems of asthma with (acute) exacerbations	0.0049
41	The average respiratory rate	0.0045
42	# of visits with same day appointments	0.0045
43	The average heart rate	0.0045
44	The maximum temperature	0.0044
45	# of asthma medication orders in the pre-index year	0.0044
46	The average SpO_2	0.0043
47	Whether the last visit's admission type is elective	0.0043
48	# of asthma reliever orders in the pre-pre-index year	0.0043
49	# of nebulizer medication orders in the pre-pre-index year	0.0043
50	Whether the patient is divorced	0.0042

51	The area's median household income	0.0042
52	Total copay for medications in the pre-index year	0.0041
53	# of days since having the last asthma diagnosis	0.0039
54	The area's percentage of household income that is between 150,000 and $199,999	0.0036
55	Whether the most emergent admission type of all of the visits is emergency	0.0035
56	# of years for which the patient had asthma	0.0033
57	Total # of units of short-acting beta-2 agonists filled	0.0031
58	Whether the patient is single	0.0029
59	The maximum percentage of blood eosinophils	0.0028
60	The maximum BMI	0.0025
61	# of asthma medication orders in the pre-pre-index year	0.0024
62	# of asthma diagnoses in the pre-index year	0.0024
63	# of diagnoses of asthma with (acute) exacerbation	0.0023
64	The proportion having asthma-related hospital encounters out of all patients with asthma in the area	0.0022
65	# of medical history diagnosis codes	0.0022
66	# of virtual visits	0.0022
67	Total # of units of asthma relievers filled	0.0022
68	# of major visits for asthma in the pre-pre-index year	0.0021
69	The relative change of weight	0.0021
70	Whether the patient is Hispanic	0.0021
71	Whether the patient is married	0.0020
72	Total # of asthma relievers ordered in the pre-pre-index year	0.0020
73	Whether the last visit's admission type is emergency	0.0019
74	Total # of days of gap in asthma controller use	0.0018
75	# of ICD-10 and ICD-9 diagnosis codes	0.0018
76	# of days since the last outpatient visit	0.0017
77	The average Z-score for weight-for-length	0.0017
78	# of days since the last use of asthma medications	0.0017
79	Total # of medications filled	0.0017
80	Whether the patient has public insurance on the last day	0.0016
81	Total # of fills of nebulizer medications	0.0016
82	The average # of days per week the patient exercises	0.0016
83	The block group's percentage of population 25 and older with college education and no degree	0.0016
84	The area's percentage of household income that is between $20,000 and $29,999	0.0015
85	# of laboratory tests with abnormal results	0.0015
86	The block group's percentage of population 25 and older with less than 9th grade education	0.0015
87	# of active problems of diabetes	0.0015
88	# of days since taking the last laboratory test	0.0015
89	The maximum systolic blood pressure	0.0014
90	The average Z-score for length-for-age	0.0014
91	Total # of units of asthma relievers filled in the pre-pre-index year	0.0014
92	# of days since the last use of short-acting beta-2 agonists	0.0013
93	Total # of units of inhaled corticosteroids filled	0.0013
94	# of days since the last use of inhaled corticosteroids	0.0013
95	# of days since the last virtual visit	0.0012
96	# of urgent care visits	0.0012
97	# of days since the last use of nebulizer medications	0.0012
98	The area's percentage of household income that is between $75,000 and $99,999	0.0011
99	Breathing abnormality like dyspnea	0.0011
100	The area's percentage of household income that is between $35,000 and $39,999	0.0011
101	# of outpatient visits	0.0011

102	# of different providers seen in outpatient visits	0.0011
103	# of days since the last other type of visit that is not an outpatient visit, an ED visit, a hospitalization, or a virtual visit	0.0011
104	# of active problems of obesity	0.0011
105	Total # of nebulizer medications ordered	0.0010
106	The area's percentage of household income that is between $10,000 and $14,999	0.0010
107	The block group's percentage of population 25 and older with 9th-12th grade education	0.0010
108	Total # of different medications ordered	0.0010
109	# of years for which the patient had chronic obstructive pulmonary disease	0.0010
110	# of patients with asthma of the PCP	0.0010
111	# of active problems	0.0010
112	# of visits	0.0009
113	# of years for which the patient had smoked based on the last record	0.0009
114	# of medical history diagnosis codes of asthma	0.0009
115	Substance use	0.0009
116	The maximum blood eosinophil count	0.0009
117	Eczema	0.0009
118	Total # of units of asthma medications filled	0.0009
119	# of active problems of anxiety/depression	0.0009
120	# of active problems of chronic obstructive pulmonary disease	0.0009
121	The average peak expiratory flow	0.0009
122	Sinusitis	0.0008
123	The maximum heart rate	0.0008
124	# of active problems of hypertension	0.0008
125	Total # of units of asthma controllers filled	0.0008
126	The area's percentage of household income that is between $30,000 and $34,999	0.0008
127	The average temperature	0.0008
128	# of days since the last ED visit	0.0008
129	The block group's percentage of population 25 and older with an associate's degree	0.0007
130	Whether the patient is a female	0.0007
131	The area's percentage of non-Hispanic black population	0.0007
132	The minimum peak expiratory flow	0.0007
133	Total # of medications ordered	0.0007
134	Among all patients with asthma of the PCP in the pre-index year, the proportion who had asthma-related hospital encounters in the index year	0.0007
135	Upper respiratory tract infection	0.0007
136	The area's percentage of household income that is between $40,000 and $44,999	0.0007
137	Total copay for asthma medications	0.0007
138	The minimum SpO_2	0.0007
139	# of active problems of asthma in the pre-index year	0.0007
140	# of active problems of atherosclerosis	0.0007
141	Chronic obstructive pulmonary disease	0.0006
142	The area's percentage of non-Hispanic population of two or more races	0.0006
143	The area's percentage of non-Hispanic American Indian and Alaska native population	0.0006
144	# of days since the last use of systemic corticosteroids	0.0006
145	The area's percentage of non-Hispanic other-race population	0.0006
146	# of cancelled appointments	0.0006
147	Whether the patient was a smoker based on the last record	0.0006
148	The area's percentage of household income that is between $50,000 and $59,999	0.0006
149	# of different medication prescribers	0.0006
150	The area's percentage of Hispanic population of two or more races	0.0006
151	The average systolic blood pressure	0.0006
152	# of active problems of rhinitis	0.0005
153	The average diastolic blood pressure	0.0005

154	Whether the patient is white	0.0005
155	The average length of stay of an ED visit	0.0005
156	The average length of appointment of an outpatient visit with asthma as the primary diagnosis	0.0005
157	# of CPT/HCPCS procedure codes	0.0005
158	The highest severity of the drug or material allergies the patient had	0.0005
159	The block group's percentage of population 25 and older with a high school diploma	0.0005
160	The area's percentage of non-Hispanic Asian population	0.0005
161	The last ED visit's length of stay	0.0005
162	# of asthma medication orders	0.0004
163	The area's percentage of Hispanic American Indian and Alaska native population	0.0004
164	The highest severity of all of the asthma diagnoses	0.0004
165	The block group's percentage of population 25 and older with a bachelor's degree	0.0004
166	Total # of oral steroid prescriptions the PCP ordered for the PCP's patients	0.0004
167	The area's percentage of non-Hispanic white population	0.0004
168	Whether the patient had any hospitalization, ED visit, or outpatient visit on asthma	0.0004
169	The area's percentage of household income that is between $125,000 and $149,999	0.0004
170	The area's percentage of Hispanic black population	0.0004
171	# of fills of systemic corticosteroids	0.0004
172	The maximum respiratory rate	0.0004
173	# of active problems of hyperlipidemia	0.0004
174	The total length of all of the hospitalizations	0.0003
175	# of active problems of asthma in the pre-pre-index year	0.0003
176	The area's percentage of household income that is <$10,000	0.0003
177	The relative change of BMI	0.0003
178	The area's percentage of household income that is between $15,000 and $19,999	0.0003
179	Pregnancy	0.0003
180	The maximum diastolic blood pressure	0.0003
181	The average length of appointment of an outpatient visit	0.0003
182	The average # of minutes per week the patient exercises	0.0003
183	Whether the patient and the PCP are of the same gender	0.0003
184	The area's percentage of Hispanic white population	0.0003
185	For the last visit, the time from making the request to the actual visit indicating the request's urgency	0.0003
186	Whether the patient used any illicit drug based on the last record	0.0003
187	Total # of antibiotic prescriptions the PCP ordered for the PCP's patients	0.0003
188	# of hospitalizations, ED visits, and outpatient visits	0.0003
189	History of bronchiolitis	0.0003
190	Total # of asthma medications filled	0.0003
191	# of fills of short-acting beta-2 agonists	0.0003
192	# of asthma medications used on the last day	0.0003
193	The average asthma control test score	0.0003
194	The average # of alcohol drinks the patient had per week across all of the records	0.0002
195	The asthma medication ratio	0.0002
196	Total # of medications ordered	0.0002
197	# of active problems of gastroesophageal reflux disease	0.0002
198	The area's percentage of Hispanic other-race population	0.0002
199	The last asthma diagnosis' severity	0.0002
200	Allergic rhinitis	0.0002
201	Cataract	0.0002
202	The area's percentage of household income that is between $45,000 and $49,999	0.0002
203	# of years for which the PCP had practiced at KPSC	0.0002
204	Whether the last hospitalization was through the ED	0.0002
205	Total # of units of systemic corticosteroids filled	0.0002

206	The area's percentage of non-Hispanic native Hawaiian or other Pacific islander population	0.0002
207	Total # of units of asthma reliever medications filled that are neither systemic corticosteroids nor short-acting beta-2 agonists	0.0002
208	Total # of opioid prescriptions the PCP ordered for the PCP's patients	0.0002
209	Total # of asthma controllers filled	0.0002
210	# of allergies of the patient	0.0002
211	# of asthma diagnoses in the pre-pre-index year	0.0002
212	The minimum post-bronchodilator FEV1/FVC ratio	0.0002
213	Indicator of environmental allergy	0.0002
214	The area's percentage of Hispanic Asian population	0.0002
215	Whether the patient speaks Spanish	0.0002
216	# of days of supply of the last asthma controller fill	0.0002
217	# of days since the last use of asthma controllers	0.0001
218	Whether the most emergent admission type of all of the visits is elective	0.0001
219	# of active problems of sleep apnea	0.0001
220	The block group's percentage of population 25 and older with a graduate or professional degree	0.0001
221	The average # of packs of cigarettes the patient smoked per day across all of the records	0.0001

Abbreviations:

BMI: body mass index
CPT: Current Procedural Terminology
FEV1: forced expiratory volume in 1 second
FVC: forced vital capacity
HCPCS: Healthcare Common Procedure Coding System
IgE: immunoglobulin E
PHQ-9: Patient Health Questionnaire-9
SpO_2: peripheral capillary oxygen saturation

References

50. Andrews AL, Simpson AN, Basco WT Jr, Teufel RJ 2nd. Asthma medication ratio predicts emergency department visits and hospitalizations in children with asthma. Medicare Medicaid Res Rev 2013;3(4):E1-10. PMID:24834366
